# Supplementary material for: MiR-106b-5p: A Master Regulator of Potential Biomarkers for Breast Cancer Aggressiveness and Prognosis
Source: Int J Mol Sci. 2021 Oct 15;22(20):11135. doi: 10.3390/ijms222011135 (PMC8539154; doi:10.3390/ijms222011135)
Supplement: Supplementary file 1 [file ijms-22-11135-s001.zip › ijms-1403078-supplementary.pdf]

Supplementary Table S1. List of target-genes used to perform Venn's Diagram.

miRNAs	Number	Common genes						
Hsa-miR-106b-5p, miR-143-3p & miR-21-5p	5	PRKAB2	TNFRSF10B	RC3H2	CBX5	STRN		
Hsa-miR-106b-5p, miR-205-5p & miR-21-5p	3	PTP4A2	GRSF1	ATL3				
Hsa-miR-106b-5p, miR-143-3p & miR-205-5p	1	AHNAK						
Hsa-miR-106b-5p & miR-21-5p	51	LTV1	GNG12	FAM160B1	KANSL1L	PHF6	ABAT	BRWD1
		CCNI	ARMC8	ZRANB1	WEE1	SESN1	MSI2	IGF2BP1
		CHD9	EPHA4	SCD	TOPORS	MAP3K2	FAM46C	RYBP
		ARL1	CNOT6	STAT3	GAPVD1	ARRDC3	NIPAL1	
		YOD1	UBE2W	HBP1	KPNA4	PLAG1	RSF1	
		PAFAH1B1	PRRX1	HIAT1	CBFB	MARCH6	GAB1	
		FRS2	YY1	PANK3	ZYG11B	DEGS1	APPL1	
		MEF2A	PPP1R3B	LAMC1	TBCEL	SNX30	RPS6KA3	
Has-miR-143-3p & miR-21-5p	14	ATXN10	GPRC5A	RMND5A	SFXN1	AP3M1	FNDC3B	RNF11
		PKD2	CCDC93	KMT2D	PDZD8	QSER1	JPH1	COL12A1
Hsa-miR-205-5p & miR-21-5p	3	FAM63B	CCNG1	TOR1AIP2				
Hsa-miR-106b-5p & miR-143-3p	21	SSH2	VGLL3	NPAT	ZNF217	ELK4	PPP2R2A	PTP4A1
		ABL2	RAD23B	PRDM4	PTPN4	VKORC1L1	CRTC3	SOBP
		PALLD	OTUD4	IER3	KAT6A	WIPF2	PPP4R2	SMIM13
Hsa-miR-106b-5p & miR-205-5p	6	ZNF827	SEMA7A	SUPT16H	B3GNT1	MED13	DNAJC13	

Hsa-miR-143-3p & miR-205-5p	1	SENP2						
Hsa-miR-21-5p	182	B3GNT5	CCAR1	SOX9	SNX29	HNRNPA2B1	ZFYVE16	DUSP16
		NUFIP2	PTK2	SEC63	FKTN	RASGRP1	CMBL	BCL11A
		ANXA1	TRIM2	RAB27B	PTAR1	EIF5	OSBPL11	TESK2
		TC2N	COPS4	FBXO46	PRICKLE2	TNPO1	CXCL10	DNM1L
		CENPQ	EIF1AX	SUB1	MTMR9	CAPN2	DDX1	ARHGAP24
		TRIM33	SKP2	PCBP1	IRF2BPL	PAPD7	IPO11	ADAM22
		HMGB3	SOS2	SMAD7	FOXK1	CEP97	AZIN1	LRRC57
		ZBTB34	THRB	DDI2	RGS1	UBA6	MDN1	CLIC2
		SMARCD1	MAPRE1	OSBPL3	RERE	ZNF10	EHD1	CLNS1A
		YEATS4	PHF16	GID4	GLCCI1	ATP5G3	RAB11A	HGSNAT
		C10orf12	NRIP1	OLR1	RTN4	OSR1	NCOA2	AGO4
		LYRM2	BNIP2	SLC44A1	FASLG	RHOB	ZNF462	GATAD2B
		ATAD2B	PRRG4	KIAA1551	FAM3C	GNAQ	NPTN	LYRM7
		TBK1	DCUN1D3	FOXN2	PRKAA2	IGF1R	AGPAT5	WDR3
		STAG2	SMC1A	PKNOX1	CCSER2	ALDH1A1	CSTB	KIAA1432
		HRK	PJA2	ZSWIM6	ATP11B	KIAA1549	MXD1	PGM3
		ARHGEF12	ENAH	TNS1	LMNB1	MRPL49	CCT5	HNRNPU
		PDCD4	LONRF2	HIPK3	KRIT1	EPM2A	RAB1A	E2F3
		DLX2	FAM208B	TXNL4A	PGRMC1	SUZ12	CUX1	GOLGA4
		ANO5	PBRM1	VPS13A	SLC31A1	MED21	RUFY3	PTPN13
		ZBTB38	LMO4	PELI1	ZNF493	ST5	MIA3	USP1
		RMND5B	KLHL9	TSPAN2	BMPR2	MED28	PCBP2	SATB1
		WHSC1L1	ZNF595	SMG1	KLF7	ESYT2	IL1B	RNF6
		SOCS6	LSM3	TSPAN31	CBX4	TMED10	HAX1	SLC16A10
		FBXO11	STK38L	SPIN1	IL10	LRRC8B	RFFL	PGK1
		ATXN7L3B	PITHD1	SNTB2	FNIP2	PCDH17	CALD1	KLF3
Hsa-miR-106b-5p	661	RRN3	SLC40A1	AHRR	FAM118A	FAM105A	MCM4	MIOS
		FSTL1	MYO5B	CCDC6	STYX	ZNF717	ZBTB10	RLIM
		USP30	RNF34	DEF8	SPRYD7	FAM102A	RAB22A	KIAA1462
		CMTR2	MZT1	ZNF724P	XPOT	ZFP91	FGF5	RAB10
		UGCG	MBTPS1	ZFHX4	MARCH7	UGDH	FGFR1OP	TMEM192
		ZHX3	DUSP2	POC1B-GALNT4	TRUB1	GPR137C	DGCR14	SFMBT1
		BTG2	PIK3C3	TNKS2	DERL2	USP31	RNF24	TXN
		OTUD1	CELF1	AFF4	SLC7A11	SLC36A1	NFATC2IP	ZNF207
		COX7A2L	LARS	SAMD10	LIPT1	SOX5	PIP4K2C	SFT2D2
		PFKP	SPOPL	AKAP10	C2orf69	RAP1B	RIT1	ZNF770
		ANKRA2	SEC23IP	ALG13	SLC5A3	ADAMTS1	NABP1	PAF1

		AEN	AGPAT3	STC1	CAMK2N1	BIRC6	GPAM	FAM117B
		FGL2	IRS1	FRMPD4	NFIC	PPP3R1	MTPN	C16orf70
		RGMB	DHX33	NSD1	UTP23	USP46	SSR1	F3
		SOS1	ZNF24	FRMD6	TES	FAM117A	SLC25A36	EIF4G2
		NHLRC3	ZZEF1	MAPRE3	WDR26	APOPT1	PPP2CA	PWWP2B
		ARHGAP11A	RAD18	ARPP19	ASB1	MED17	C2CD2	FICD
		G3BP1	RAI14	LDLR	C3orf70	NLK	KIF13A	EPAS1
		MPZL2	FKBP14	RIF1	BCL11B	BCORL1	NAA30	CDC5L
		UBE2G1	FGD4	COPS2	RP2	EID2B	MSMO1	UBE3C
		AGFG1	DYNC1LI2	BVES	UBE2B	FMNL3	ADIPOR1	HNRNPH2
		GTF2A1	CRIPT	RBM26	CCNT2	SNAP23	SKIDA1	MKRN1
		EP300	COIL	VLDLR	JOSD1	TMED8	GCNT4	NCOA7
		RASSF3	GPATCH2L	DBT	RNF138	SDHAF2	SYBU	ZBTB5
		C2orf49	APPBP2	HPS3	SLC35A1	PIP4K2A	DDX17	MORF4L1
		NBEAL1	ZBTB18	NAA50	LASP1	ARHGAP1	KLHDC10	PEX5L
		KBTBD8	COA5	SAMD9L	PLAA	ZBTB33	PKIA	SBNO1
		AP1G1	TMEM33	NTN4	ZBTB4	MED7	SRSF2	DOCK7
		GNAI3	ARIH1	EPHA7	MBD2	ZNF706	RAB12	B4GALT5
		VCPIP1	SIPA1L2	EDNRB	HEG1	S1PR1	ZNF106	TRPS1
		NETO2	ERAP1	EIF2S1	ZBTB6	CIC	ABHD10	FBXO28
		SLC16A14	ARL4C	EPRS	MCL1	TMX3	MED12L	CASP2
		MMS22L	POU6F1	TNFRSF17	EHBP1	MTURN	ABHD17B	CAPN7
		DSP	CCNG2	RBM7	WDR82	ANKRD13C	PANK2	BNC2
		IRF1	SCD5	SPAG9	PRNP	CHD2	ACVR1B	GTF2H3
		INIP	ACTR1A	ZNF264	FAF2	JMY	DCAF10	RANBP2
		SOX4	VPS25	PDPK1	CEP104	SLC9A6	TM9SF3	ETV1
		C5orf24	AQP4	CD2AP	MAN1A2	SNX9	FAM83D	EOGT
		PTPDC1	UBN2	WDR36	USP37	DCUN1D1	SMG7	TTC9
		MED13L	TANC1	MYCL	PTBP1	POLR2F	GINM1	ADAMTS5
		NFIA	NEBL	ROBO1	FBXO30	REEP5	SLC41A1	STK38
		RAB8B	GPATCH2	XIAP	CSRNP3	FOXA1	TGFBR2	CYBRD1
		CCDC50	UBXN7	TMEM9B	CDC40	TSC22D2	C16orf87	RBP1
		ATG2B	TP53INP2	SLC36A4	GALNT10	IFI44L	ACAP2	PPP2R3A
		TAF7	ANTXR2	GPR176	H1F0	RASA2	ZNF680	BMP2K
		LAPTM4A	PCCB	GNS	CREBL2	ANKFY1	SUCO	C3orf38
		RFC3	MAK16	IFNAR1	BBX	ABHD13	GXYLT1	SESN2
		ARL4A	SMYD5	MKLN1	CREB5	IQGAP1	NBL1	HNRNPL
		TAOK3	TPBG	LAMTOR5	WFS1	CCPG1	KCNN2	TMEM56
		RUNX3	TUSC2	B2M	PTDSS1	CKAP2	FBXL3	TMEM168
		SLK	SUV420H1	KIF5A	CUL1	TMEM181	TMEM30A	FJX1
		DIP2C	GMFB	TBC1D20	DEPDC1	ZBTB9	ATXN1	NARS

		ZNF202	TCF4	PARD6G	TNFAIP1	ITPKB	FAM168A	RNF128
		MAP3K5	NUPL1	ZFYVE20	ZDHHC17	FGF2	CTNND1	PHTF2
		ROCK2	POGK	ADO	PKN2	SLAIN2	GPD2	CSNK2A1
		ZNF704	NRAS	ZNF362	SEC23A	AMMECR1 L	ZNF800	EPM2AIP1
		CYP2U1	CXCL8	UBE2J1	MCF2L	SKI	VANGL1	WAC
		SQSTM1	MAPK9	TGFBR1	LGALSL	TET2	PDZD11	LRPAP1
		FOXK2	UTP14C	HAS2	VASH2	CCNT1	CPS1	SIK1
		TMEM127	PPTC7	WDR4	DCTN4	LAMTOR1	CCDC71L	FCHO2
		SAMD12	CSTF2T	CNOT7	BRI3BP	TCERG1	CCND2	RUFY2
		LNPEP	RPP14	CRIM1	KCNK2	RRM2	LZIC	SLC17A5
		CTDSPL2	KIF14	CCND1	SOGA1	PHACTR2	AJUBA	STK17B
		KPNA3	37469	TCEB3	SIKE1	KLHL8	PIK3R1	HIPK1
		FBXO22	CDR2	NOTCH2	VEZF1	ARAP2	CYB561D1	ARSJ
		KCNC1	FOXJ2	TSNAX	PRKAR2B	PRR15	ANKIB1	MBOAT2
		FITM2	DUSP18	KDM6B	KDM5B	MFN1	HELZ	PARD6B
		GOLGA3	C10orf76	PSMD12	DNM3	ANKRD50	EIF4A2	FMN2
		BTG1	AHR	ZBTB7A	TCF7L1	EFNB2	SCAMP2	PDE4A
		C5orf28	MAGT1	IPO5	CITED2	IPO7	TET1	SAR1B
		MFSD8	COQ10B	UBE2Q2	CLSTN1	RNASEH2B	PREPL	ZNF850
		RAPH1	SERTAD2	KLF12	MYLIP	F2R	CENPI	PAK2
		PCYT1A	GOLGA1	PLEKHO2	RBM12	CAMSAP2	KLHL20	VHL
		MSH6	IQSEC2	KCNK1	NUCKS1	RBBP7	SNRPD3	MAP3K9
		GREB1	C7orf60	MEX3D	EIF4E	PIGN	PPM1G	UBXN2A
		PELO	DYRK2	CHMP3	PRPF4	DNAJC27	SPRY4	SMAD5
		MOSPD2	PCNX	IPMK	MKNK2	TMBIM6	RAP2C	C1orf43
		REEP3	RNF2	GPR157	DEDD	TNFRSF21	FBXO48	CAMK2N 2
		POLR1E	SH3PXD2 A	AGFG2	FOXJ3	ZNF367	RORC	RBMS1
		SEMA3A	ABCD2	FAM199X	RCCD1	PDE7A	OTULIN	ZNF445
		BTN3A1	PGM2L1	CDCA7	SLC35F5	RASL11B	RNF149	MDM4
		RPA2	ANKRD52	IRS2	EZH1	TIMMDC1	WDR37	STT3B
		TRIM3	ARNTL2	NAPEPLD	DVL3	SMU1	CDC42SE2	SYAP1
		LAMP3	TAGAP	CRYBG3	METTL14	RSBN1	SH3RF1	C2CD4A
		ABCA1	PFN2	SPECC1	F13A1	SMOC1	VMA21	UBC
		REST	TMPO	CBX8	PTPRD	KIAA1191	HIVEP3	NDN
		PLXNA1	MOB1B	CUL3	IL17RD	MOB1A	ARHGAP3 5	DUSP8
		OTUD3	C6orf62	RND3	TFAM	GSPT1	CAPN15	DCAF8
		LRRC58	RRAS2	CALCOCO 2	SASH1	C5orf30	RIMKLA	DNAJC10
		PPM1A	NDUFS2	TRIB1	SLC22A23	CNTNAP2	ASF1A	SLC39A6
		IPO8	STRIP1	ZKSCAN1	GIPC2	TMEM200C	CTSA	XRCC2
		ULK1	NRBP1	YPEL1	LDLRAP1	MACC1	TTPAL	

		CAMTA1	ASXL2	SLC48A1	FAM8A1	KCMF1	ZMPSTE24	
		SLC2A3	BLOC1S5-TXNDC5	ITGB8	KIAA0247	SOCS1	SKIL	
		TEAD3	ZNF738	PHF12	USP15	AP3D1	CERCAM	
Hsa-miR-143-3p	133	CD44	CCSER1	GATAD1	FAM60A	TPP1	CRAMP1L	37104
		CIPC	LIMD1	KIAA1147	XPR1	LIFR	ARSD	KLHL11
		RAE1	CHD6	PTGS2	PRKCE	HCCS	EIF2AK1	COL5A1
		C9orf91	KIAA0355	NPR3	PTPN23	MGA	MAP1B	VTI1B
		NUP155	INO80D	SIPA1L3	INPP5F	PACS1	RANBP10	JDP2
		ZDHHC2	SARS	ADAM10	SLC25A25	FSCN1	RARG	ARCN1
		TFDP2	CPSF7	ANKRD17	EIF2AK2	TRAK2	BRWD3	AKAP11
		GPR180	PDP1	DRAM1	TMEM178B	CMPK1	KANK2	ZSCAN22
		BBC3	ATF7	ATXN7	GFPT1	SECISBP2L	SLC1A3	MTMR3
		TMEM19	VASH1	CTH	R3HCC1L	CXCL12	REL	GATM
		CAND1	MED14	TMED5	WDR11	EPG5	LOXL4	PGD
		TENM4	HSPA4L	CSNK2A2	SAP130	TMEM245	YARS	SLC38A7
		COL1A1	ASH1L	PPIA	KRT80	TBL1XR1	AMOTL1	RPL37
		PTPN11	EMP1	MLLT3	ARHGAP18	THBS1	RGP1	GGA2
		GTF3C3	ARPC5	GPD1L	MAPK1	JARID2	37865	SF3B3
		C5orf38	ZNF709	AMIGO1	UBE2R2	CA2	HIPK2	VPS45
		ZFP37	CDK6	KPNA1	IAPP	PRSS23	TCF20	AMER1
		FNDC1	TMEM248	IGFBP5	ANKH	PAFAH1B2	AKAP8	HDLBP
		SRSF11	ZNF281	SLC25A42	ETFDH	DCP2	IGF2R	SENP5
Hsa-miR-205-5p	48	C9orf78	KIFAP3	TTC3	CBWD5	HNRNPH3	CHCHD3	SRPR
		PTEN	BCL2	NDUFA4	NXF1	SEH1L	CCNJ	PSMD13
		NDST1	FEM1B	CBX1	VEGFA	CRLF3	HSPA9	UPP1
		TERF2IP	MRPS17	PSMD2	TP53BP1	SNW1	CREB1	CHURC1
		CHMP5	TSPYL4	RNF168	TCEAL4	IRF4	TDG	CALM1
		NEFH	E2F1	APTX	RAB31	ROCK1	TMEM39B	PHLDB2
		PSMG4	MYL6	KCNJ10	YES1	LETM2	PRELID1	

Supplementary Table S2. Target genes and their pathways for hsa-miR-205-5p and miR-143-3p.

Term	P-value	Adjusted P-value	Genes
**Disease**	1,13E-07	8,75E-05	SEH1L;PSMD13;SRPR;PTEN;PSMD2;MAPK1;RPL37;RAE1;KPNA1;SUPT16H;IGFBP5;NUP155;PRKCE;GFPT1;EIF2AK2;ADAM10;PTPN11;CREB1;PACS1;IAPP;TPP1;CALM1;GRSF1;PPIA;CD44;DCP2
**p53 activity regulation**	1,08E-05	0,003653404	CDK6;CCNG1;BCL2;PTEN;E2F1;TNFRSF10B;THBS1;BBC3;HIPK2

Signaling by PDGF	1,41E-05	0,003653404	COL1A1;CREB1;COL5A1;PRKCE;PTEN;MAPK1;PTPN11;CALM1;THBS1
Senescence and autophagy	2,20E-05	0,004045415	COL1A1;IGFBP5;BCL2;PTEN;E2F1;MAPK1;THBS1;CD44
Integrins in angiogenesis	2,61E-05	0,004045415	COL1A1;ROCK1;COL5A1;COL12A1;MAPK1;PTPN11;VEGFA
**HIV factor interactions with host**	1,37E-04	0,013408765	SEH1L;NUP155;PSMD13;PACS1;PSMD2;PPIA;RAE1;KPNA1
Signaling events mediated by PRL	1,38E-04	0,013408765	PTP4A1;PTP4A2;ROCK1;MAPK1
**Gastrin pathway**	1,60E-04	0,013408765	BCL2;PTGS2;CD44;IER3;VEGFA
**Interleukin-2 signaling pathway**	1,72E-04	0,013408765	YARS;YES1;AHNAK;PRKCE;DNAJC13;PTPN11;PKD2;MLLT3;PRSS23;BBC3;VEGFA;PTP4A1;RAB31;INPP5F;ATXN7;IRF4;REL;BCL2;MAPK1;UPP1;PTPN4;PHLDB2;IER3
MicroRNA regulation of DNA damage response	1,73E-04	0,013408765	CREB1;CDK6;CCNG1;E2F1;TNFRSF10B;BBC3
**Physiological and pathological hypertrophy of the heart**	2,27E-04	0,015990105	PRKCE;MAPK1;LIFR;CALM1
**Rev-mediated nuclear export of HIV-1 RNA**	2,64E-04	0,017048987	SEH1L;NUP155;RAE1;KPNA1
**Interleukin-5 regulation of apoptosis**	3,08E-04	0,017633183	YES1;CREB1;BCL2;REL;UPP1;PTGS2;CD44;IER3
**Type I interferon (interferon-alpha/beta) pathway**	3,25E-04	0,017633183	PTP4A1;PTP4A2;PTPN11;PTPN23;PTPN4
Signaling by EGFR in cancer	3,41E-04	0,017633183	CREB1;PRKCE;PTEN;ADAM10;MAPK1;PTPN11;CALM1
**Melanocyte development and pigmentation pathway**	4,20E-04	0,019338708	CREB1;BCL2;MAPK1
**Transport of mature transcript to cytoplasm**	4,63E-04	0,019338708	NXF1;SEH1L;NUP155;RAE1;SRSF11
**Gene expression**	4,70E-04	0,019338708	YARS;RARG;SUPT16H;CPSF7;GTF3C3;SEH1L;SF3B3;NUP155;PSMD13;SRPR;SARS;PPP2R2A;NXF1;MED14;SNW1;MED13;TFDP2;TBL1XR1;PSMD2;RPL37;RAE1;ZFP37;DCP2;SRSF11
**Developmental biology**	4,73E-04	0,019338708	SEMA7A;ROCK1;CSNK2A2;PTPN11;COL1A1;MYL6;MED14;MED13;CREB1;COL5A1;TBL1XR1;ABL2;MAPK1;IAPP
**Hypothetical network for drug addiction**	5,16E-04	0,020017683	CREB1;PRKCE;MAPK1;TERF2IP
**HIV infection**	6,19E-04	0,022858136	SUPT16H;SEH1L;NUP155;PACS1;PSMD13;PSMD2;RAE1;PPIA;KPNA1
**PDGFB signaling pathway**	8,40E-04	0,025987802	YES1;PRKCE;PTEN;EIF2AK2;MAPK1;PTPN11;ARPC5
**Interferon signaling**	8,56E-04	0,025987802	SEH1L;NUP155;IRF4;EIF2AK2;PTPN11;RAE1;CD44;KPNA1
**Activation of BH3-only proteins**	9,64E-04	0,025987802	BCL2;E2F1;BBC3
**Glioma**	9,99E-04	0,025987802	CDK6;PTEN;E2F1;MAPK1;CALM1

**Cyclin D-associated events in G1**	0,001002263	0,025987802	CDK6;TFDP2;E2F1;PPP2R2A
**EGFR interaction with phospholipase C-gamma**	0,001002263	0,025987802	CREB1;PRKCE;MAPK1;CALM1
**Transport of mature mRNAs derived from intronless transcripts**	0,001002263	0,025987802	NXF1;SEH1L;NUP155;RAE1
**p38 alpha/beta MAPK downstream pathway**	0,001002263	0,025987802	ELK4;CREB1;CSNK2A2;PTGS2
**Tight junction**	0,001004683	0,025987802	YES1;PRKCE;CSNK2A2;PTEN;PPP2R2A;AMOTL1;ASH1L
**Capped intron-containing pre-mRNA processing**	0,00124565	0,031181422	NXF1;CPSF7;SEH1L;SF3B3;NUP155;RAE1;SRSF11
**Signaling by ERBB2**	0,001300078	0,031526902	YES1;CREB1;PRKCE;PTEN;MAPK1;CALM1
**Activation of NOXA and translocation to mitochondria**	0,001343031	0,031581588	E2F1;BBC3
**Antiviral mechanism by interferon-stimulated genes**	0,001396186	0,031669421	SEH1L;NUP155;EIF2AK2;RAE1;KPNA1
**Bladder cancer**	0,001465087	0,031669421	E2F1;MAPK1;THBS1;VEGFA
**Influenza infection**	0,0014692	0,031669421	SEH1L;NUP155;EIF2AK2;RPL37;GRSF1;RAE1;KPNA1
**Estrogen receptor signaling pathway**	0,001574974	0,032926757	CREB1;BCL2;MAPK1
**Axon guidance**	0,001676784	0,032926757	COL1A1;MYL6;SEMA7A;CXCL12;CREB1;COL5A1;ROCK1;CSNK2A2;ABL2;MAPK1;PTPN11
**Interactions of HIV Vpr protein with host cellular proteins**	0,001744653	0,032926757	SEH1L;NUP155;RAE1;KPNA1
**RANKL regulation of apoptosis and immune response**	0,001789221	0,032926757	MYL6;BCL2;ADAM10;CALM1;CD44
**Focal adhesion**	0,001801245	0,032926757	COL1A1;MYL6;COL5A1;ROCK1;BCL2;PTEN;MAPK1;THBS1;VEGFA
**S1P/S1P1 pathway**	0,001821634	0,032926757	MAPK1;PTGS2;VEGFA
**Immune system signaling by interferons, interleukins, prolactin, and growth hormones**	0,00182455	0,032926757	SEH1L;YES1;NUP155;IRF4;EIF2AK2;MAPK1;PTPN11;RAE1;CD44;KPNA1
**Syndecan 1 pathway**	0,002059214	0,036317054	COL1A1;COL5A1;COL12A1;MAPK1
**Integrated breast cancer pathway**	0,002165805	0,037348099	CREB1;BCL2;PTEN;E2F1;MAPK1;HIPK2;VEGFA
**Signaling by SCF-KIT**	0,002257788	0,038087894	CREB1;YES1;PTEN;MAPK1;PTPN11
**Apoptosis**	0,002328336	0,03844232	ROCK1;PSMD13;IRF4;PSMD2;BCL2;E2F1;TNFRSF10B;BBC3;KPNA1
**Inactivation of BCL-2 by BH3-only proteins**	0,002776877	0,044892848	BCL2;BBC3
**Small cell lung cancer**	0,003119812	0,049407642	CDK6;PTEN;E2F1;BCL2;PTGS2

Supplementary Table S3. Target genes and their pathways for hsa-miR-21-5p and miR-106b-5p.

Term	P-value	Adjusted P-value	Genes
**Interleukin-2 signaling pathway**	1,28E-07	1,39E-04	RERE;TES;BTG1;CXCL8;IRS1;CITED2;FASLG;IRS2;SLC2A3;AHR;PKD2;GALNT10;FGF5;RPS6KA3;CCND2;CCND1;RGS1;CIC;IER3;IL10;EPHA4;CSNK2A1;BTN3A1;VPS13A;ARAP2;RRAS2;VEZF1;DUSP8;JOSD1;TGFBR2;ITPKB;PTP4A1;IL1B;IRF1;WAC;SOS1;FAM8A1;B4GALT5;USP15;AHNAK;CBFB;PLAG1;PIK3R1;TXN;RASGRP1;MAPK9;NRAS;SOCS1;CAMK2N1;STK38;UBC;S1PR1;ZSWIM6;E2F3;ZBTB7A;LDLR;CDR2;FKTN;SPAG9;STAT3;RAB27B;DNAJC13;SMAD5;EHD1;CENPI;PKIA;CCNG2;STK17B;NFIC;YPEL1;PDCD4;ESYT2;PTPN4;BRWD1;PDE7A
**TGF-beta signaling pathway**	7,36E-06	0,004023598	MEF2A;PDPK1;STAT3;CUL1;XIAP;PPP2R2A;PIK3R1;RUNX3;SMAD5;PTK2;TGFBR1;SMAD7;TGFBR2;PPP2CA;SKI;PPM1A;MAPK9;ZFYVE16;CCND1;EP300;SIK1;SOS1;SKIL;PAK2
**T cell receptor regulation of apoptosis**	1,14E-05	0,00416079	BTG1;CXCL8;POGK;FASLG;YY1;TBK1;CCND2;CCND1;CASP2;CEP97;IER3;IL10;ANXA1;F2R;ATP11B;VEZF1;DUSP8;RAD23B;ZDHHC17;PPM1A;HAX1;TMX3;RP2;IL1B;IRF1;KIAA0247;TFAM;TMBIM6;MAPRE1;IFNAR1;PSMD12;EPAS1;SATB1;CUL1;SLC5A3;RASGRP1;NRAS;PGK1;BNIP2;ZBTB7A;MCL1;ZBTB18;MAP3K2;ERAP1;TNFRSF10B;KLHL20;CXCL10;SCD;PKIA;STK17B;JMY;PDE7A;TMEM9B
**MAPK signaling pathway**	1,63E-05	0,004464616	FASLG;NLK;ACVR1B;FGF2;RASGRP1;DUSP16;ELK4;FGF5;RAP1B;RPS6KA3;MAPK9;PPP3R1;NRAS;MKNK2;CASP2;MAP3K9;PAK2;EIF4E;MAP3K5;MEF2A;MAP3K2;DUSP2;RRAS2;GNG12;DUSP8;TGFBR1;TGFBR2;PPM1A;TAOK3;IL1B;RASA2;SOS1;SOS2
**Messenger RNA processing**	3,52E-05	0,006916179	RANBP2;TMED10;CELF1;DDX1;HNRNPU;CSTF2T;GTF2H3;SMC1A;CDC40;CCAR1;PPM1G;HNRNPL;PRPF4;PTBP1;PCBP1;PCBP2;HNRNPA2B1;SRSF2;POLR2F;HNRNPH2;SNRPD3;TXNL4A;NUPL1;EIF4E
**Signaling by FGFR mutants**	3,80E-05	0,006916179	FGF5;NRAS;CUX1;STAT3;GAB1;FGFR1OP;FRS2;PIK3R1;SOS1;FGF2
**Messenger RNA splicing: major pathway**	7,43E-05	0,010071272	HNRNPL;PTBP1;PRPF4;PCBP1;PCBP2;HNRNPA2B1;SRSF2;HNRNPU;HNRNPH2;SMC1A;CDC40;CCAR1
**Antigen presentation: folding, assembly, and peptide loading of class I MHC proteins**	8,23E-05	0,010071272	PSMD12;SEC23A;RNF34;UBE3C;SAR1B;UBA6;CUL3;CUL1;RNF6;UBE2J1;SOCS1;RNF138;UBE2Q2;UBC;VHL;SKP2;B2M;UBE2B;ERAP1;LNPEP;UBE2G1;PJA2;KLHL20;KLHL9;UBE2W;MKRN1;ASB1

**Eukaryotic protein translation**	8,75E-05	0,010071272	EIF4A2;EIF5;EIF1AX;EIF2S1;EIF4E;EIF4G2
**Capped intron-containing pre-mRNA processing**	9,21E-05	0,010071272	RANBP2;HNRNPU;SMC1A;CDC40;CCAR1;HNRNPL;PRPF4;PTBP1;PCBP1;PCBP2;HNRNPA2B1;SRSF2;POLR2F;HNRNPH2;SNRPD3;TXNL4A;EIF4E;NUPL1
**Neurotrophic factor-mediated Trk receptor signaling**	1,03E-04	0,010227585	RAP1B;NRAS;RIT1;CCND1;MCF2L;STAT3;GAB1;FRS2;PIK3R1;SOS1;SQSTM1
**Protein processing in the endoplasmic reticulum**	1,18E-04	0,010775865	MBTPS1;SEC23A;SAR1B;WFS1;CUL1;DERL2;YOD1;UBE2G1;EIF2S1;RAD23B;UBE2J1;MAPK9;MAN1A2;DNAJC10;CAPN2;PLAA;SSR1;STT3B;MAP3K5;SEC63
**Insulin signaling pathway**	1,37E-04	0,010973012	PRKAA2;IRS1;ROCK2;SNAP23;RORC;IRS2;PIK3R1;IGF1R;RPS6KA3;MAPK9;NRAS;SOCS1;PRKAR2B;MKNK2;MAP3K9;EIF4E;MAP3K5;MAP3K2;PRKAB2;CSNK2A1;PDPK1;GAB1;RHOB;EHD1;PPP1R3B;PIK3C3;SOS1;SOS2
**p53 activity regulation**	1,41E-04	0,010973012	DYRK2;RRM2;TNFRSF10B;FBXO11;YY1;PPP2CA;MAPK9;CCND2;CCND1;SESN1;CCNG2;SESN2;CCNG1;EP300;MDM4;SKP2
**Signaling by insulin receptor**	1,90E-04	0,013310553	PRKAB2;PRKAA2;IRS1;PDPK1;GAB1;IRS2;FRS2;PIK3R1;FGF2;FGF5;PPM1A;NRAS;PIK3C3;SOS1;EIF4E
**eIF4E and p70 S6 kinase regulation**	2,01E-04	0,013310553	EIF4A2;PPP2CA;IRS1;PDPK1;PIK3R1;EIF4E;EIF4G2
**Wnt signaling pathway and pluripotency**	2,07E-04	0,013310553	TCF7L1;CTNND1;PPP2R2A;ZBTB33;PPP2R3A;NLK;PPP2CA;MAPK9;CCND2;CCND1;EP300;DVL3;LDLR;PAFAH1B1
**Skeletal muscle hypertrophy is regulated via AKT/mTOR pathway**	2,43E-04	0,014326993	PPP2CA;PDPK1;PIK3R1;EIF2S1;EIF4E;IGF1R
**Signaling by TGF-beta receptor complex**	2,49E-04	0,014326993	SKI;PPM1A;USP15;CCNT2;CCNT1;UBC;SKIL;TGFBR1;TRIM33;TGFBR2;SMAD7
**Gene expression**	2,75E-04	0,015038801	EIF4A2;SMG1;ZNF493;CCNT2;THRB;CCNT1;CHD9;RORC;HNRNPU;PPP2R2A;EPRS;SMG7;MED17;CCAR1;NARS;PTBP1;MED13;SNRPD3;ZNF445;TXNL4A;TNPO1;ZNF202;TEAD3;NCOA2;SUPT16H;EIF1AX;ZNF10;SMC1A;CDC40;NRBP1;MED7;PPM1A;PRPF4;CLNS1A;SRSF2;TFAM;HNRNPH2;ZNF595;NOTCH2;GTF2A1;PSMD12;RRN3;PPP2CA;LARS;PCBP1;PCBP2;UBC;TCEB3;SSR1;POLR2F;EIF4E;SKIL;ZKSCAN1;RANBP2;ZNF264;MBD2;GTF2H3;

			EIF2S1;LSM3;SMAD7;SKI;HNRNPL;CNOT6;EIF5;CNOT7;NFIA;NFIC;HNRNPA2B1;NUPL1;TRIM33
**SHP2 signaling**	3,07E-04	0,015734837	NRAS;IRS1;LMO4;GAB1;GNAI3;FRS2;PIK3R1;SOS1;ARHGAP35;IGF1R
**IGF1 pathway**	3,26E-04	0,015734837	IRS1;PDPK1;IRS2;PIK3R1;SOS1;PTK2;IGF1R
**Cyclin D-associated events in G1**	3,38E-04	0,015734837	PPP2CA;CCND2;CCND1;UBC;CUL1;PPP2R2A;E2F3;SKP2
**Signaling by NGF**	3,46E-04	0,015734837	MEF2A;ARHGEF12;PRDM4;IRS1;PDPK1;STAT3;FRS2;IRS2;PIK3R1;RTN4;PPP2CA;DNM3;RPS6KA3;FGD4;NRAS;RIT1;PRKAR2B;MCF2L;UBC;CASP2;SOS1;SQSTM1;SOS2
**E-cadherin nascent AJ-like junctions pathway**	4,07E-04	0,017565651	RAP1B;ENAH;CCND1;CSNK2A1;CTNND1;IQGAP1;PIK3R1;KLHL20
**Interleukin-4 regulation of apoptosis**	4,18E-04	0,017565651	CXCL8;CITED2;SATB1;FGL2;F13A1;OSBPL11;RASGRP1;ATXN1;S1PR1;CASP2;KCNN2;TNS1;IL10;ANXA1;F3;ZDHHC17;ITPKB;ARL4C;CCPG1;STK17B;RASA2;MCM4;RBMS1;MXD1;SOS1;FJX1
**Developmental biology**	4,54E-04	0,018363952	SEMA7A;ROCK2;CHD9;SEMA3A;NTN4;LAMC1;ACVR1B;ARHGAP35;MED17;ROBO1;RPS6KA3;NRAS;MED13;ABL2;PLXNA1;EP300;BNIP2;PAK2;MEF2A;SPAG9;NCOA2;PRNP;ARHGEF12;CSNK2A1;RGMB;PTK2;MED7;MED13L;RHOB;ENAH;DNM3;MED21;TCF4;SOS1;SOS2;PFN2
**PRC2 complex long-term gene silencing through modification of histone tails**	4,75E-04	0,018544146	SUZ12;YY1;CBX4;RBBP7;EZH1
**Apoptosis**	5,27E-04	0,019302118	HRK;DSP;PSMD12;SATB1;XIAP;TNFRSF10B;FASLG;PIK3R1;PTK2;IGF1R;LMNB1;PPP3R1;H1F0;PRKAR2B;IL1B;IRF1;UBC;CAPN2;CASP2;DNM1L;PAK2;TNFRSF21;APPL1;MCL1
**Signaling by bone morphogenetic protein (BMP)**	5,59E-04	0,019302118	SKI;BMPR2;ZFYVE16;FSTL1;SMAD5;SMAD7
**Plasma membrane estrogen receptor signaling**	5,82E-04	0,019302118	NRAS;ROCK2;GNAQ;GNAI3;STRN;PIK3R1;SOS1;IGF1R
**Transcriptional activity of SMAD2/SMAD3-SMAD4 heterotrimer**	5,82E-04	0,019302118	SKI;PPM1A;CCNT2;CCNT1;UBC;SKIL;TRIM33;SMAD7
**Endocytosis**	5,83E-04	0,019302118	IQSEC2;F2R;ZFYVE20;ARAP2;PARD6G;RAB22A;RAB11A;TGFBR1;IGF1R;SMAD7;TGFBR2;EHD1;DNM3;ACAP2;

			PARD6B;ZFYVE16;CHMP3;LDLRAP1;DNM1L;VPS25;LDLR
**Signaling by Robo receptor**	6,21E-04	0,01994998	ENAH;ABL2;SOS1;PAK2;SOS2;ROBO1;PFN2
**Immune system**	6,44E-04	0,020098146	EIF4A2;UBE3C;IRS1;IRS2;RPS6KA3;ACTR1A;TBK1;UBE2Q2;AP1G1;KIF5A;EP300;CASP2;KPNA4;ARIH1;SKP2;KPNA3;B2M;MEF2A;PDPK1;ZFYVE20;PJA2;ENAH;DNM3;DYNC1LI2;SIKE1;IL1B;IRF1;PELI1;PIK3C3;SOS1;SQSTM1;IFNAR1;SEC23A;PSMD12;RNF34;SAR1B;UBA6;DCTN4;CUL3;CUL1;RNF6;PIK3R1;TXN;RASGRP1;UBE2J1;RAP1B;PPP2CA;MAPK9;NRAS;SOCS1;RNF138;PCBP2;UBC;VHL;EIF4E;PAK2;CTSA;RANBP2;UBE2B;STAT3;ERAP1;LNPEP;UBE2G1;KLHL20;KLHL9;UBE2W;MKRN1;NUPL1;EIF4G2;ASB1
**BMP receptor signaling**	6,89E-04	0,020923874	SKI;PPM1A;BMPR2;ZFYVE16;XIAP;RGMB;SMAD5;SMAD7
**FSH regulation of apoptosis**	7,60E-04	0,022284792	RAB1A;SMARCD1;CTNND1;SNAP23;ARHGAP1;MSMO1;CREBL2;VLDLR;NLK;ARHGAP35;PPP3R1;PGRMC1;PGK1;LDLR;IER3;MAP3K5;CSNK2A1;BCL11A;MBD2;RHOB;TGFBR2;ARL4C;PTP4A1;GPRC5A;TMBIM6
**p75 neurotrophin receptor-mediated signaling**	7,75E-04	0,022284792	ARHGEF12;PRDM4;XIAP;PIK3R1;RTN4;RHOB;FGD4;MAPK9;MCF2L;UBC;NDN;CASP2;SOS1;SQSTM1;SOS2
**TGF-beta regulation of extracellular matrix**	8,69E-04	0,024111626	DLX2;SEC23A;CITED2;SEMA3A;FGL2;SLC40A1;STC1;AHR;PIK3R1;SLC7A11;LAMC1;UGCG;GOLGA4;PGRMC1;CCND1;ADAMTS1;STK38;S1PR1;SSR1;HAS2;BNIP2;E2F3;FAM3C;SKIL;MAP3K5;MAP3K2;MBTPS1;TCF7L1;F2R;ZNF10;PTPN13;F3;SMAD7;SKI;ARL4A;WEE1;NFIA;SCD;IL1B;STK17B;RBP1;CCNG1;SOS2;PFKP
**mTOR signaling pathway**	9,04E-04	0,024111626	EIF4A2;PRKAA2;RRN3;IRS1;PDPK1;PIK3R1;YY1;PPP2CA;RPS6KA3;NRAS;PDCD4;ULK1;EIF4E;EIF4G2
**HIV-1 elongation complex formation in the absence of HIV-1 Tat**	9,04E-04	0,024111626	SUPT16H;CCNT2;CCNT1;TCEB3;GTF2H3
**Signaling by FGFR in disease**	0,001077714	0,027434015	PDPK1;STAT3;GAB1;FRS2;PIK3R1;IL17RD;FGF2;FGF5;PPP2CA;NRAS;CUX1;PRKAR2B;UBC;FGFR1OP;SOS1
**EGF receptor (ErbB1) signaling pathway**	0,001095619	0,027434015	NRAS;STAT3;GNAI3;GAB1;PIK3R1;SOS1;PTK2
**Actin cytoskeleton regulation**	0,001126934	0,027434015	ARHGEF12;ROCK2;F2R;RRAS2;IQGAP1;PIK3R1;GNG12;SSH2;FGF2;PTK2;ARHGAP35;ENAH;FGF5;NRAS;PIP4K2A;ITGB8;PIK3C3;PIP4K2C;SOS1;PAK2;SOS2;PFN2

**TGF-beta signaling in development**	0,001129488	0,027434015	BMPR2;ACVR1B;SMAD5;TGFBR1;SMAD7;TGFBR2
**Nuclear beta-catenin signaling and target gene transcription regulation**	0,001331625	0,031640577	NCOA2;TCF7L1;CXCL8;CCND2;CCND1;CUL1;IGF2BP1;EP300;DVL3;HBP1;TCF4
**Fatty acid, triacylglycerol, and ketone body metabolism**	0,001461916	0,033661437	ABCA1;AGPAT5;NCOA2;PRKAB2;PRKAA2;CHD9;AGPAT3;MED7;MED13L;MED17;MED13;GPAM;GPD2;MED21;PCCB;EP300;TNFRSF21;TEAD3
**ERBB1 downstream pathway**	0,001494205	0,033661437	MAP3K2;PDPK1;STAT3;GAB1;PPP2R2A;IQGAP1;PIK3R1;PPP2CA;RPS6KA3;MAPK9;NRAS;CAPN2;SOS1
**IGF-1 receptor and longevity**	0,001570662	0,033661437	IRS1;PDPK1;PIK3R1;SOS1;IGF1R
**PTEN-dependent cell cycle arrest and apoptosis**	0,001570662	0,033661437	PDPK1;FASLG;PIK3R1;SOS1;PTK2
**Signaling by constitutively active EGFR**	0,001570662	0,033661437	NRAS;UBC;GAB1;PIK3R1;SOS1
**Renal cell carcinoma**	0,001622071	0,034094692	RAP1B;NRAS;EPAS1;GAB1;EP300;PIK3R1;VHL;SOS1;SOS2;PAK2
**Axon guidance**	0,001725157	0,035577285	SEMA7A;ROCK2;SEMA3A;GNAI3;NTN4;LAMC1;ARHGAP35;ROBO1;EFNB2;RPS6KA3;PPP3R1;NRAS;ABL2;PLXNA1;PAK2;EPHA4;PRNP;EPHA7;ARHGEF12;CSNK2A1;RGMB;PTK2;RHOB;ENAH;DNM3;SOS1;SOS2;PFN2
**Ubiquitin-mediated proteolysis**	0,001988086	0,040240326	UBE3C;UBE2B;UBA6;CUL3;CUL1;XIAP;UBE2G1;UBE2J1;KLHL9;UBE2W;SOCS1;UBE2Q2;BIRC6;VHL;SKP2
**Signaling events regulated by Ret tyrosine kinase**	0,002126068	0,042250776	IRS1;GAB1;IRS2;FRS2;PIK3R1;SOS1;PTK2
**FGF signaling pathway**	0,002179446	0,04253812	PDPK1;CTNND1;STAT3;GAB1;FRS2;PIK3R1;IL17RD;SOS1;FGF2
**Interleukin-1 signaling pathway**	0,002396851	0,045920259	MAP3K2;CXCL8;UBE2B;CUL1;UBE2G1;PIK3R1;UBE2J1;ELK4;UBE2W;MAPK9;UBE2Q2;IL1B;PELI1;SQSTM1
**Signaling events mediated by focal adhesion kinase**	0,002444606	0,045920259	RAP1B;MAPK9;CCND1;ROCK2;CAPN2;PIK3R1;SOS1;ARHGAP35;PTK2

**Signaling by FGFR1 mutants**	0,00247877	0,045920259	FGF5;CUX1;STAT3;FGFR1OP;PIK3R1;FGF2
**IGF1 signaling pathway**	0,002540664	0,046264285	CSNK2A1;IRS1;PIK3R1;SOS1;IGF1R
**Neurotrophin signaling pathway**	0,002581996	0,046264285	PRDM4;IRS1;GAB1;IRS2;FRS2;FASLG;PIK3R1;RAP1B;RPS6KA3;MAPK9;NRAS;SOS1;SOS2;MAP3K5
